# Liberté

**DOI:** 10.1186/s12978-022-01471-1

**Published:** 2022-07-29

**Authors:** Natasha Amartey, Bassey Ebenso

**Affiliations:** 1 Illinois, USA; 2 Leeds Institute of Health Sciences, University of Leeds, Leeds, UK

- Committee for the Promotion of Virtue
- And the Prevention of Vice
- Oh, how we godly entice
- Nonreligious saints
- To become
- Depraved,
- Degenerate
- Like us.

- Force life not yet born
- Massacre
- Once they can
- Hear, smell, taste, see
- Experience
- Unconscionable evil
- No Deity can ever
- “Make beautiful in its time.”

- To profess 1791’s Amendment II
- To the Constitution of the
- White man’s U.S. of A.:
- The right to arm teens, or racists, or…
- With semi-automatics
- Maximizing gruesome
- Deaths per second with
- The Law

- Tobacco products’ sale
- To under age 21
- Is a
- Crime.
- Purchase of guns
- At age 18
- Not only legal,
- But sublime.

- Pro-Life, you have fought the good fight
- You have won
- The Right to abort
- The Living, Thinking, Loving lives of
- Children and adults
- In the false security of
- Schools, stores, theaters, no less
- Save the tools of mass murder;
- Collateral bloodshed
- Not our mess!

- Victims identifiable
- Solely by DNA
- The human form
- Pulverized
- By your lawful
- Weapons
- Of Mass
- Destruction

- Wasn’t that
- The justification
- For America
- Invading
- A sovereign nation? And yet
- We aim
- These same WMDs
- At our child- and minority populations.

- I do solemnly swear…
- 1) To support and defend
- The Constitution
- Against all enemies foreign
- (Domestic be exempt)
- 2) That I will bear
- True faith and allegiance
- To the rights of man:

- The Right of males
- To impregnate, molest, rape
- The assortment of females:
- Adults, teens, or children
- 525 billion sperm cells per male
- Populate—unrestricted—the accursed earth
- Prosecution as likely
- As governments structurally enforcing Female Worth

- Pro-Life victorious in
- The Right of males
- To abandon
- Inseminated females
- And resulting offspring
- Subjecting such to
- Hardship, poverty, abuse
- Unremitting generations of
- Oppression
- For the Man’s Right to Choose

- Abortion abolitionists?
- Since when did being God-fearing
- Become synonymous with
- Sanctity
- of Hate?
- Eliminating women’s autonomy and rights
- Insufficient.
- Men alone shall determine her fate.

- Inconceivable conception
- Audaciously absent:
- National debate about
- Accountability of
- The Sperm
- …
- Or did we women
- Rape ourselves too?

- Submission for scholarly admonition:
- “Omit the profanity.”
- *Then omit the profane!*
- *The unjust, the inhumane!*
- How does protection of a
- Blastocyte, embryo, or fetus
- Nullify the protection of an
- Impregnated woman or child?

- Authorizing ownership of AR-15s and AK-47s
- Pro-Life, how many more innocents
- Must be sacrificed at your altar?
- Authorizing State ownership of female bodies
- Your stranglehold on women’s and children’s uteri
- Does this prove you believe in God?
- “Even demons believe that—and shudder!”

- America! America!
- God shed His grace on thee
- Our founding fathers
- Deaf to Native Americans’ and Africans’ plea
- And crown thy good
- With the likelihood
- That minorities, targets of semi-auto slaughter
- And females
- Shall never truly be
- Equal, autonomous and free.

~ *Natasha Amartey*^*1*^* and Bassey Ebenso*^*2*^

*Note: this poem speaks of the coercion and abuse of females by males due to the potential for impregnation and abandonment that might occur, in order to comment on abortion rights being struck down in the United States. In no way do the authors infer that such force, coercion and abuse is solely carried out by males on females. We acknowledge that all genders are capable of abusing any gender.

